# Accumulating Evidence for Dance/Movement Therapy in Cancer Care

**DOI:** 10.3389/fpsyg.2018.01778

**Published:** 2018-09-28

**Authors:** Sharon W. Goodill

**Affiliations:** Department of Creative Arts Therapies, College of Nursing and Health Professions, Drexel University, Philadelphia, PA, United States

**Keywords:** dance/movement therapy, review, cancer care, RCT, qualitative

## Abstract

This review offers a discussion of the state of dance/movement therapy (DMT), research with people living with cancer. The vast majority of extant studies published in the English language are with women with breast cancer. An examination of challenges facing DMT researchers in this area is provided, and recommendations for research foci, and priorities are outlined. These include qualitative syntheses, integration of implementation evidence with qualitative synthesis, and formal process evaluation studies.

## Introduction

This review aims to offer an update about research on dance/movement therapy (DMT) as a psychosocial support intervention in the context of cancer care, for both adult, and pediatric populations. The literature search was conducted with a university based web-scale discovery service housing 651 databases including PsycInfo, Alt HealthWatch, Medline, ProQuest, and CINAHL. It intersected subject terms dance therapy, DMT, and dance movement psychotherapy with cancer, psycho-oncology, and oncology, in a publication timeframe from 1960 to December 2017. The search was neither exhaustive nor systematic, yet attempted completeness within these parameters: (1) the publication was identified as research including RCTs, CCTs, and one group pre-posttest designs, using quantitative, qualitative, or mixed methods designs; (2) Full text was available in the English language; (3) Descriptions of interventions were sufficient for this author to determine if the intervention was consistent with the professional practice of DMT worldwide. This included evidence of professional training, and education of the interventionist. The determination of this third criterion may have been biased by the perspective of the author, a United States based DMT educator with 30 years of experience preparing graduate level dance/movement therapists in a university setting, plus international teaching. The sample also included studies discovered through hand searching reference lists. Projects that were research question driven, and reported systematic data collection, and analysis were considered research works. Program descriptions with vignettes, case examples, or anecdotal quotations from patients only, and basic narrative case reports were not. Studies on interventions which were reported as dance only (taught as a class, vs. conducted as a therapeutic session), were excluded. Of the 79 reports discovered in the search, 34 were excluded because they were not research reports; 20 were excluded because the intervention was not named as either DMT, dance therapy, or dance movement psychotherapy, or the intervention was judged as inconsistent with professional DMT practice; 4 were not published in English, and 9 were not on cancer care. After removing two duplicates, 10 studies (represented in 11 publications) remained for review. As a contributor to the 2015 Cochrane Collaboration systematic review of DMT for people with cancer (Bradt et al., 2015), I have drawn from that publication, and acknowledge here the excellent work by co-authors Joke Bradt, Ph.D., MT-BC, and Minjung Shim, Ph.D., BC-DMT.

## Dance/Movement Therapy in Cancer Care

As a mind/body integrated form of psychotherapy, DMT combines the benefits of dance, movement, emotional expression, social support, and creative activity in a single intervention approach. Koch and Fischman (2011), described how DMT is an embodied, enactive approach, in which patients learn to translate sensory, and affective cues into cognitions, verbalizations, and new behaviors. Behavioral engagement is built into the treatment, which is structured to help patients explore, try, and learn new ways of expressing, responding, and communicating. The body level learning that occurs in this embodied, enactive work means that newly learned patterns could be easily generalized into everyday life.

Conducted by trained, credentialed professionals, this occurs in context of a supportive psychotherapeutic relationship, and is informed by clinical assessment, which includes movement observation [typically based in the Laban Movement Analysis framework (Cruz, 2013; Serlin, 1999, Unpublished)]. Clinical methods reported in the literature on DMT in cancer care include body awareness techniques, rhythmic action with musical accompaniment, non-verbal, and verbal expression of emotions, relaxation methods (often with imagery), improvisation, and the use of symbolic, and metaphoric communication in movement. Treatment goals, and outcomes measured in published studies on DMT in cancer care include overall quality of life, stress management, and perceived stress, pain management, reduction of anxiety, and fatigue, increases in sense of vitality, and energy, body awareness, and body image, social support, and the recognition of need for support, self-efficacy, and improved self-care, meaning making, and increases in resilience, and the installation of hope. The DMT is usually offered in group therapy formats, which adds the common factors associated with all group interventions, e.g., mutual support, and lessening the sense of isolation. Further, DMT shares several common components with other complementary, and integrative health disciplines. These have been identified as follows: a whole person focus that attends to social, mental, physical, and spiritual dimensions; a therapy that is a formal discipline with education, credentialing, and ethical standards; takes an optimal health focus that is wellness oriented; is evidence informed (considering patient preferences, cultural, and clinical state along with the best available research evidence); combines with conventional care rather than positioning as alternative; uses a patient-centered approach, and gives import to the patient-provider relationship (Rosenthal and Lisi, 2014).

## Review of Studies

Ten studies are presented in this mini-review. There are four RCTs, three controlled clinical trials, and four pre-experimental studies (with one group pretest-posttest designs). Nine are with adults, and eight of these specifically with women living with breast cancer. One is with children, and one included family caregivers of adult cancer patients.

Across all studies, the DMT offered included a combination of both movement, and verbal expression. Two offered DMT integrated with other creative arts therapies (CAT), and while most delivered the DMT in group formats, two (Madden et al., 2010; Goldov, 2012), provided individual therapy. Other variations in the DMT clinical methods used in this collection of studies may have been rooted in cultural differences, and preferences. For example, the studies by Ho and colleagues were conducted in Hong Kong with Chinese participants, and included culturally congruent elements along with the Western based DMT structures. The Sharma study offered culturally responsive DMT sessions with an ethnically mixed sample including Alaska Native people. Dance/movement therapists often integrate other movement forms into sessions, and this is true in the Sharma (2016) and Sandel et al. (2005), studies which both integrated aspects of The Lebed Method (Healthy Steps Program, n.d.), Dibbel-Hope employed the Authentic Movement method (Pallaro, 1999), for her intervention.

In this collection of studies, there is a mix of participant samples that included people in active treatment, and those who had completed treatment. Qualitative data were collected in several of these studies, and yet true mixed method data integration was generally not conducted. The three RCTs with women with breast cancer (Dibbel-Hope, 2000; Sandel et al., 2005; Ho et al., 2016a), were the only studies included in the Bradt et al. (2015) Cochrane review, and yet all of the other studies shown herein had been examined for eligibility in that review, and several were covered in other recent reviews (Boehm et al., 2014; Koch et al., 2014; Archer et al., 2015; Serlin et al., 2017). For this reason, **Table 1** below does not reiterate findings from each but instead shows other main features of studies, to inform recommendations.

**Table 1:** Features of several DMT studies in cancer care.

Author(s), year ^∗^	Sample	Design^∗∗^	Treatment/dosage
Dibbel-Hope, 2000	*N* = 31 (analyzed) Women w/ Breast cancer	RCT	3 h. sessions, 1/week x 6 weeks.
Sandel et al., 2005	*N* = 37 (analyzed) Women w/ breast cancer	RCT	1 h. group sessions, 2/weeks x 6 weeks. plus1/week x 6 weeks.
Ho et al., 2016a	*N* = 139 (analyzed) Women w/ breast cancer	RCT	1.5 h. group sessions, 2/weeks x 3 weeks.
Ho et al., 2016b	*N* = 104 Women w/ breast cancer	QUAL arm of Ho, et al. RCT	1.5 h. group sessions, 2/weeks x 3 weeks.
Madden et al., 2010	*N* = 16, Children w/ brain tumors, all genders	MM, RCT	1 h. sessions, 3/week. of CAT, including DMT
Goldov, 2012	*N* = 14, Women w/ breast cancer	CCT	Five individual sessions over 2 weeks.
Serlin et al., 2000	Women w/ breast cancer	MM, One group pretest-posttest w/ QUAL	Group DMT, 2 h. sessions, 1/week x 12 weeks.
Mannheim and Weis, 2006	*N* = 77 Women, all cancers	MM, One group pretest-posttest design	Group DMT, 90 min. sessions, 2 or 3/weeks. for average of 7 sessions.
Sharma, 2016	*N* = 16, All genders, all cancers, plus their caregivers	MM, CBPR	Open group DMT, 90–120 min. sessions, 1/week × 12 weeks.
Klagsbrun et al., 2005	*N* = 18, Women w/ breast cancer	One group pretest-posttest design	2 day workshop, 14 h. total, expressive therapies incl. DMT
Ho, 2005	*N* = 16, Women w/ breast cancer	One group pretest-posttest design	Group DMT, 90 min. sessions 1/week x 6 weeks.


*^∗^See reference list for full publication information. ^∗∗^RCT, Randomized Controlled Trial; CCT, Controlled Clinical Trial; MM, mixed methods; CBPR, Community Based Partner Research; QUAL, qualitative study or phase of study.*

## Discussion and Recommendations

The 2015 Cochrane Collaboration review included the three RCTs with adult oncology populations, follows the Cochrane standards for review criteria, and concluded that according to those criteria there is not sufficient evidence to support claims of effectiveness at this time (Bradt et al., 2015). However, it was also noted that in those RCTs, DMT was well tolerated, with small dropout rates (Bradt et al., 2015). This indicates acceptability of the therapy, and bodes well for future studies with larger samples and more rigorous designs.

In addition, qualitative findings from various studies (e.g., Dibbel-Hope, 2000; Serlin et al., 2000; Ho et al., 2016b) suggest that there are patient perceived benefits, and improvements in several aspects of QOL which were not captured in the statistical analyses. These are findings generated by careful analysis of systematically collected narrative data, usually through semi-structured interviews. Recently, the Cochrane Collaboration has promoted not only qualitative synthesis as a form of systematic review, but also guidelines for integrating implementation evidence, and qualitative synthesis in the context of systematic reviews on intervention effectiveness (Hardena et al., 2018). A qualitative synthesis from studies on DMT in cancer care, and a review that integrates the quantitative, and qualitative outcomes, using the guidelines put forth by Hardena et al. (2018) is recommended. Such a review could yield new, and better targeted foci for future RCTs by identifying the outcome variables that DMT is most likely to impact. In addition, careful process evaluation (Moore et al., 2015) could elucidate the causative assumptions in DMT for those living with cancer, and reveal possible mechanisms of change for this therapy in the oncology context.

Research published to date is hindered by several factors that could be addressed with increased coordination between researchers. Published studies have all provided good descriptions of the DMT provided, and as noted above, these reveal considerable variability in how the DMT is delivered. The range of methods used by dance/movement therapists in practice is indeed very broad (American Dance Therapy Association [ADTA], 2017), and the creative patient-centered nature of the therapy means that context, and patient preferences will always drive clinical reasoning to a large degree. Nonetheless, the use of manualized protocols which retain the context-sensitive and improvisational aspects of practice have been piloted, and are possible in CAT research (Rolvsjord et al., 2009). It is recommended that future studies employ protocols that would permit replications. Another limitation in the research to date is that that many were designed, and reported as pilot studies, but larger follow up studies were not conducted, or reported. This is not uncommon with studies conducted as doctoral projects (e.g., Dibbel-Hope, 2000; Goldov, 2012; Sharma, 2016). At present, there are very few research faculty positions for DMT worldwide, and this puts constraints on both the depth, and volume of research studies conducted in any single clinical area. A notable exception to this for studies in cancer care is at the University of Hong Kong, where Dr. Ho and colleagues have built a robust research program with several studies in the intersection of psycho-oncology, mind/body dynamics, DMT, and the other CAT. One challenge to researchers is related to the fact that, at least in the United States at present, many DMT clinical services to people and families living with cancer is funded with hourly contracts, through philanthropy, or in training internships. This is true of other complementary, and integrative therapies, but does create an unstable clinical environment in which to conduct trials. Nonetheless, a search of the American Dance Therapy Association member directory indicates that 13% of credentialed members of the association reported specializing in either medical DMT, or palliative care, indicating both professional interest, and increasing use of DMT in contexts relevant to cancer care.

## Conclusion

This review of studies on DMT in cancer care has several limitations. The use of English language publications only is a notable limitation. For example, at least one clinical trial (Mannheim et al., 2013; a MM CCT with *N* = 115), was omitted. Others have recently published reviews of dance, DMT, the CAT, or arts interventions in cancer care (Boehm et al., 2014; Archer et al., 2015), each with slightly different inclusion criteria, levels of completeness, use of meta-analytic statistics and ways of evaluating quality of evidence. Another limitation of this review emanates from an inconsistency among included studies, and that is that not all of them reported ethnic, and/or racial characteristics of the study samples. It is important that future publications always include this information so that the influence of culture on approaches to treatment, choices of research methodologies, and interpretation of results can be fully informed.

This is an evolving discussion in a dynamic healthcare environment that is increasingly open to complementary therapies and the importance of embodied approaches.

## Author Contributions

SG is the sole author, and responsible for all material in this mini-review.

## Conflict of Interest Statement

The author declares that the research was conducted in the absence of any commercial or financial relationships that could be construed as a potential conflict of interest.
